# Sex differences in brain aging among adults with family history of Alzheimer’s disease and *APOE4* genetic risk

**DOI:** 10.1016/j.nicl.2021.102620

**Published:** 2021-03-10

**Authors:** Sivaniya Subramaniapillai, Sricharana Rajagopal, Jamie Snytte, A. Ross Otto, the PREVENT-AD Research Group, Gillian Einstein, M. Natasha Rajah

**Affiliations:** a Department of Psychology, McGill University, 2001 Avenue McGill College, Montréal, QC H3A 1G1, Canada; b Brain Imaging Centre, Douglas Institute Research Centre, 6875 LaSalle Blvd Verdun, Montréal, QC H4H 1R3, Canada; c Department of Psychology, University of Toronto, 100 St. George Street, Toronto, ON M5S 3G3, Canada; d Rotman Research Institute, Baycrest Hospital, 3560 Bathurst St, Toronto, ON M6A 2E1, Canada; e Tema Genus, Linköping University, TEMA-huset, Entrance 37, Room E433, Campus Valla, Linköping, Sweden; f Department of Psychiatry, Faculty of Medicine, McGill University, 1033 Avenue des Pins, Montréal, QC H3A 1A1, Canada

**Keywords:** Family history, *APOE*, Brain aging, Alzheimer’s disease

## Abstract

Emerging evidence suggests that Alzheimer’s Disease (AD) risk factors may differentially contribute to disease trajectory in women than men. Determining the effect of AD risk factors on brain aging in women, compared to men, is critical for understanding whether there are sex differences in the pathways towards AD in cognitively intact but at-risk adults. Brain Age Gap (BAG) is a concept used increasingly as a measure of brain health; BAG is defined as the difference between predicted age (based on structural MRI) and chronological age, with negative values reflecting preserved brain health with age. Using BAG, we investigated whether there were sex differences in the brain effects of AD risk factors (*i.e.*, family history of AD, and carrying an apolipoprotein E ε4 allele [*+APOE4*]) in cognitively intact adults, and if this relationship was moderated by modifiable factors (*i.e.* body mass index [BMI], blood pressure and physical activity). We undertook a cross-sectional study of structural MRIs from 1067 cognitively normal adults across four neuroimaging datasets. An elastic net regression model found that women with a family history of AD and *+APOE4* genotype had more advanced brain aging than their male counterparts. In a sub-cohort of women with those risk factors, higher BMI was associated with less brain aging whereas lower BMI was not. In a sub-cohort of women and men with *+APOE4*, engaging in physical activity was more beneficial to men’s brain aging than women’s. Our results demonstrate that AD risk factors are associated with greater brain aging in women than men, although there may be more unexplored modifiable factors that influence this relationship. These findings suggest that the complex interplay between unmodifiable and modifiable AD risk factors can potentially protect against brain aging in women and men.

## Highlights

• +*APOE4* women with family history of AD had greater brain aging than men.
• Non-modifiable risk factors interact with modifiable ones to decrease brain aging.
• Higher BMI was associated with less brain aging in *+APOE4* women.
• Sex differences in *APOE* status on brain aging were found in physical activity.
• Greater physical activity was associated with less brain aging in *+APOE4* men.

## 1 Introduction

Women represent two-thirds of the cases of late-onset Alzheimer’s Disease (AD; Alzheimer’s Association, 2019, Bailly et al., 2019, Mielke et al., 2014, Nebel et al., 2018, Schmidt et al., 2008). Indeed, female biological sex constitutes a risk to AD, after accounting for sex differences in longevity (Buckley et al., 2019, Prince et al., 2016). Moreover, women with an apolipoprotein E ε4 allele (*+APOE4* genotype) exhibit greater AD pathology than men, particularly in relation to tau pathology (Buckley et al., 2019, Hohman et al., 2018, Ramanan et al., 2019), demonstrating that this genetic risk factor confers greater AD risk in women, compared to men (Altmann et al., 2014, Breitner et al., 1999, Bretsky et al., 1999, Farrer et al., 1997, Mortensen and Hogh, 2001, Payami et al., 1996).

More sensitive measures for detecting early signs of AD in individuals at risk of the disease are actively investigated. One such measure is an individual’s brain age gap (BAG), defined as the difference between predicted brain age and chronological age. Brain age is derived from a prediction model that estimates a participant’s ‘brain age’ from their individual MRI data (Franke and Gaser, 2019). Thus, BAG is a neuroimaging marker of brain health, representing how much an individual varies from a normative aging trajectory. A negative BAG reflects preserved brain health in the face of aging and is associated with higher educational attainment (Steffener et al., 2016), and healthy lifestyle factors, such as physical activity (Steffener et al., 2016) and meditation (Luders et al., 2016). Conversely, a positive BAG reflects decrements in brain health in the face of aging and is associated with poorer physical fitness (Cole et al., 2018), diabetes (Franke et al., 2013), and increased mortality risk (Cole et al., 2018). A positive BAG is also linked with dementia, in that some studies show that BAG is a more accurate predictor of conversion from Mild Cognitive Impairment (MCI) to AD than other traditional metrics, including cognitive scales and CSF biomarkers (Gaser et al., 2013, Löwe et al., 2016). One study associated higher baseline BAG with greater risk of AD conversion, independent of *APOE* status (Löwe et al., 2016). Therefore, there is growing evidence for the predictive power of BAG for conversion to AD. However, to our knowledge no study to date has explicitly examined the effect of sex on BAG, and its interaction with AD risk factors (i.e., family history of AD [+FH], and *+APOE4* genotype).

In the current study, we used MRIs to train a regularized (Elastic Net) regression model to predict age from brain structure in a cognitively normal cohort. The regularization technique employed mitigates against overfitting and is robust to extreme correlations among predictor variables (Friedman et al., 2010, Kragel et al., 2012). One recent study demonstrated that compared to other prediction models (e.g., Random Forest, Kernel Ridge Regression) across many cohort and feature sizes, the elastic net model was the most flexible and accurate regression technique for neuroimaging data (Jollans et al., 2019). Further, elastic net models have also been shown to have comparable performance to relevance vector regressions, another commonly used model in predicting age from MRI data (Jonsson et al., 2019, Lee et al., 2020).

Since we do not know exactly when AD-related changes occur, particularly in a cognitively normal healthy cohort, we used data to capture the entire adult lifespan. Aging studies typically model participants across the adult lifespan particularly since older adulthood might not capture slight but significant differences happening earlier on in midlife, a life stage that has received little attention (Madan, 2017) but is becoming increasingly important to investigate since AD-related changes can start as soon as midlife or earlier in younger adulthood (Reiman et al., 2004, Ritchie et al., 2015, Rajah et al., 2017). In addition, the lifelong presence of +FH or *APOE* risk factors justified our inclusion of participants from young to older adulthood so that our prediction model would be sensitive to age-related differences throughout the adult lifespan. Since we do not know exactly when AD-related changes occur, particularly in a cognitively normal healthy cohort, a model that reflects the adult lifespan would therefore make no *a priori* assumptions of when the consequences of AD risk factors may emerge throughout the lifespan.

We applied this trained model to MRIs in a separate cohort of cognitively normal adults with a known (FH+) and unknown (uFH) family history of AD to predict participants’ brain age. We tested for sex differences in the effect of FH status and *APOE* genotype on BAG. We hypothesized that AD risk factors (e.g., FH+ and *+APOE4*) would contribute to a more positive BAG (i.e., advanced brain aging relative to the expected age-normative trajectory) in women, compared to men. We conducted secondary analyses to determine if cardiovascular and lifestyle (modifiable) factors (i.e., BMI, Blood Pressure, and Physical Activity) moderated the effect of AD risk factors on BAG in women, compared to men. We hypothesized that a healthy BMI and greater physical activity would be associated with a more negative BAG. This is the first study to date to consider whether biological sex modulates the association between AD unmodifiable risk factors (i.e., FH+ and *APOE-e4*) and BAG.

## 2 Material and methods

### 2.1 Datasets

T1-weighted (T1w) MRIs were used from four neuroimaging datasets: Dallas Lifespan Brain Study (DLBS), South Asian Lifespan Dataset (SALD), Montreal Memory and Aging Lifespan Study (MMALS), and Pre-symptomatic Evaluation of Experimental or Novel Treatments for Alzheimer’s Disease (PREVENT-AD). We used open-access datasets when possible because they allowed us to use large cohort sizes for the train and test sets, which yields greater model accuracy (Jollan et al., 2019). We chose these four neuroimaging datasets specifically because they represent a cognitively healthy and well-educated cohort of participants across the adult lifespan. Since the prevalence of women with AD varies slightly according to geography (Rizzi et al., 2014), it was also important that we pick datasets across diverse geographic locations (Canada, USA, China) to reflect this variation in AD risk. Finally, the MMALS and PREVENT-AD datasets have information about the FH and *APOE* status of participants. Both these datasets were also collected at the Douglas Mental Health University Institute (Montreal) so there would be no differences in scanning site in the post-hoc analyses that were conducted on these specific datasets.

Sex was determined by self-report in the MMALS and PREVENT-AD cohorts, but the method of assessment was not indicated for the DLBS and SALD cohorts. The total cross-sectional lifespan cohort after minimal quality control (QC) consisted of 1067 cognitively healthy adults (18–89 years old; 697 females, 370 males). In the unknown FH (uFH) cohort, datasets either included participants with a known negative family history of AD (MMALS) or unreported FH Status (DLBS, SALD), with mixed *APOE* status. A positive family history of AD (i.e., +FH) was defined as a parental or multiple sibling history of late onset sporadic AD (Tremblay-Mercier et al., 2020, Rajah et al., 2017 for more details). FH status was obtained using the Cache County Study questionnaire (Hayden et al., 2009, Tschanz et al., 2006). *APOE* status for three datasets (DLBS, MMALS, PREVENT-AD) was determined through genotyping of venous blood samples (see Supplementary Table 1 for detailed information on genotyping procedures).

We randomly selected 70% of uFH participants to train the age-prediction model, while the remaining 30% of the uFH cohort were combined with the +FH cohorts from the PREVENT-AD and MMALS cohorts to test the age-prediction model. The model was trained on a uFH cohort so that model-estimated predictive effects would be computed relative to the uFH healthy aging trajectory, while the test set contained both the uFH and +FH participants so that we could directly estimate the effect of +FH status on BAG. See Table 1 for summary of participant demographics in the train and test sets (see Supplementary Appendix and Supplementary Table 2 for participant demographics and MRI parameters for each dataset).

**Table 1:** Demographic details of the train and test sets.

	Train	Test 1: uFH*	Test 2: +FH
n	596	251	220
Age	47.39 (18.46)	47.50 (19.15)	61.53 (6.04)
Sex	218 (37%) M, 378 (63%) F	90 (36%) M, 161 (64%) F	62 (28%) M, 158 (72%) F
Age Group	224 (38%) YA, 186 (31%) MA, 186 (31%) OA	95 (38%) YA, 78 (31%) MA, 78 (31%) OA	73 (33%) MA, 147 (67%) OA
TIV (cm^3^)	1056.83 (103.81)	1047.50 (108.44)	1011.75 (95.31)
Age Group: Male	72 OA, 60 MA, 84 YA	30 OA, 25 MA, 35 YA	46 OA, 16 MA
Age Group: Female	114 OA, 126 MA, 140 YA	48 OA, 53 MA, 50 YA	101 OA, 57 MA

TIV = Total Intracranial Volume; M = Male, F = Female, YA = Younger Adult (19–39 years), MA = Middle-aged (40–59 years), OA = Older Adult (60–89 years). Values in brackets are in standard deviation. *The uFH cohort comprise of three datasets with reported negative FH Status (MMALS) or unreported FH Status (DLBS, SALD).

The DLBS cohort (Dallas, USA) consisted of 275 healthy adults (21–89 years; 175 females, 100 males). The SALD cohort (Chonqing, China) is publicly available data (Wei et al., 2018), which consisted of 433 participants (19–80 years; 272 females, 161 males). Data from DLBS (http://fcon_1000.projects.nitrc.org/indi/retro/dlbs.html) and SALD (http://fcon_1000.projects.nitrc.org/indi/retro/sald.html) cohorts were made available by International Neuroimaging Data-sharing Initiative (Mennes et al., 2013) and hosted on Neuroimaging Informatics Tools and Resources Clearinghouse (Kennedy et al., 2016). The MMALS cohort (Montréal, Canada) was collected in Dr. Rajah’s lab (Ankudowich et al., 2016 for further details), and consisted of 169 participants (19–76 years; 116 females, 53 males). Thirty participants from the MMALS cohort were known to have a +FH for AD (43–58 years; 24 females, 6 males) so were only used in the test set. The MRI dataset is not currently openly available due to Institutional Ethics Regulations, but the demographics data are available (https://github.com/sivaniya/brain_age). The study protocol for the MMALS cohort was approved by the ethics board at the Faculty of Medicine at McGill University. All participants in the cohort provided their informed consent to participate in the study.

The PREVENT-AD cohort (Montréal, Canada) (Tremblay-Mercier et al., 2020) data release 5 was used in the current study. Some of the MRI data used from the PREVENT-AD are openly available at https://openpreventad.loris.ca/. However, in the current analyses, data release 5 MRI and *APOE* data were used. All participants included in the current study were cognitively normal (CDR = 0 indicating no dementia) and had parental or multiple-sibling history for AD. The cohort, which consisted of 190 individuals (55–80 years; 134 females, 56 males), was solely used as an independent test set.

### 2.2 T1w MRI pre-processing

The prediction model specifically focused on cortical thickness measures and subcortical regions because these measures are sensitive to age-related differences and are also expected to produce high model prediction accuracy (Cole, 2020). These measures were obtained from T1w MRIs acquired on 3T scanners (Supplementary Table 2 for details on imaging sites). Images were first pre-processed using the minc-bpipe-library pipeline (https://github.com/CobraLab/minc-bpipe-library), and then submitted to the CIVET 2.1.0 (https://mcin.ca/technology/civet/) pipeline to obtain cortical thickness measurements. The measurements for each participant were parcellated into 64 ROIs using the Desikan-Killiany-Tourville (DKT) atlas (Klein and Tourville, 2012, Collins et al., 1994, Lerch and Evans, 2005). Fourteen subcortical volumes were extracted from the aseg parcellation generated by FreeSurfer version 6.0 (Fischl et al., 2002) (see Supplementary Appendix for QC methodological details and Supplementary Table 6 for complete list of 82 features used).

### 2.3 Brain age prediction using elastic net regression

#### 2.3.1 Model training

We first used a sex-stratified model training approach by training two elastic net models: one for women only, and one for men only. Both models performed similarly (Supplementary Fig. 1), which motivated our use of a mixed-sex training model to leverage the larger cohort size in both the training and testing sets. We present the methods and results for the mixed-sex cohort below.

To build the elastic net model, the uFH cohort (from the DLBS, SALD, and MMALS datasets) were first split into training and testing sets using the createDataPartition function in the caret package (Kuhn, 2008) in R, which randomly split the cohort by Age and Sex into 70% training (n = 596) and 30% testing (n = 251; Table 1). In order to have a proportional representation of women and men (i.e., Sex) across the adult lifespan (i.e., Age) in the train and test sets, the age of participants was coded into three age bins: younger adults (YA:19–39 years), middle-aged adults (MA: 40–59 years) and older adults (OA: 60–89 years). These age groups were closely adapted from previous studies that investigated participants throughout the adult lifespan (Ankudowich et al., 2016, Grady et al., 2006, Kwon et al., 2016, Subramaniapillai et al., 2019, Subramaniapillai et al., 2018). The dot product of Age Group and Sex was then used to split the participants so that the train and tests in the uFH cohort were represented proportionally according to Age Group and Sex. See Table 2, Table 3, Table 4 for a more detailed summary of participants in the full cohort, train, and test sets, respectively.

**Table 2:** Detailed demographic details of the full cohort.

FULL COHORT (n = 1067)	DLBS	SALD	MMALS	PREVENT-AD
n	275	433	169	190
Age	53.30 (19.86)	43.81 (17.23)	47.87 (16.20)	63.10 (4.60)
Men	53.59 (20.02)	44.70 (17.67)	45.81 (17.32)	63.61 (5.03)
Women	53.13 (19.82)	43.28 (16.98)	48.81 (15.65)	62.89 (4.40)
Sex	175F; 100 M	272F; 161 M	116F; 53 M	134F; 56 M
TIV (cm^3^)	1049.71(106.87)	1064.28 (101.18)	1032.26 (109.82)	1007.48 (92.82)
Education (years)	16.41 (2.35)	–	15.59 (2.11)	15.69 (3.36)
% of +FH (for the available cohorts)	–	–	17.80%	100%
*APOE* status distribution	n = 228	–	n = 166	n = 190
e2/e2	3		0	0
e2/e3	22		11	14
e3/e3	143		104	107
e4/e2	8		6	6
e4/e3	49		40	58
e4/e4	3		5	5
BMI (kg/m^2^)	–	–	24.36 (3.68); n = 168	22.20 (3.86)
MOCA	–	–	28.93 (1.13)	28.03 (1.54); n = 189
MMSE	28.37 (1.27)	MMSE ≥ 25	29.28 (0.92)	–
Systolic BP	–	–	118.11 (15.50); n = 108	129.99 (16.52)
Diastolic BP	–	–	74.56 (7.59); n = 108	75.28 (8.99)
Physical Activity	–	–	–	n = 180
Low Exercise				103
High Exercise				77

TIV = Total Intracranial Volume; +FH = positive family history of AD; BMI = Body Mass Index; MOCA = Montreal Cognitive Assessment; MMSE = Mini Mental State Examination; BP = Blood Pressure; M = Male; F = Female. Note: Education level is obtained as the total number of years of education in the DLBS and PREVENT-AD cohorts, and total number of years in proportion to the degree obtained for the MMALS cohort. Values in brackets are in standard deviation. Information that was not collected/reported by the datasets are marked with a dash. Summary information is presented for the full cohort, unless the cohort size is listed.

**Table 3:** Detailed demographic details of the train set.

TRAIN SET (n = 596)	DLBS	SALD	MMALS
n	190	310	96
Age	52.96 (19.41)	43.85 (17.28)	47.82 (17.61)
Men	52.62 (19.61)	44.48 (17.65)	47.31 (18.31)
Women	53.16 (19.38)	43.46 (17.09)	48.04 (17.44)
Sex	119F; 71 M	192F; 118 M	67F; 29 M
TIV (cm^3^)	1054.75 (102.18)	1067.24 (101.79)	1027.31 (108.51)
Education (years)	16.39 (2.23)	–	15.77 (2.16)
% of +FH (for the available cohorts)	–	–	0%
*APOE* Status (count)	n = 158	–	n = 95
e2/e2	0		0
e2/e3	15		8
e3/e3	99		63
e4/e2	4		3
e4/e3	37		18
e4/e4	3		3
BMI (kg/m^2^)	–	–	23.94 (3.52); n = 95
MOCA	–	–	28.85 (1.15)
MMSE	28.28 (1.27)	MMSE ≥ 25	29.44 (0.82)
Systolic BP	–	–	118.18 (15.50); n = 65
Diastolic BP	–	–	74.54 (6.78); n = 65

TIV = Total Intracranial Volume; +FH = positive family history of AD; BMI = Body Mass Index; MOCA = Montreal Cognitive Assessment; MMSE = Mini Mental State Examination; BP = Blood Pressure. Note: The PREVENT-AD was solely used in the Test Set. Education level is obtained as the total number of years of education in the DLBS and PREVENT-AD cohorts, and total number of years in proportion to the degree obtained for the MMALS cohort. Values in brackets are in standard deviation. Information that was not collected/reported by the datasets are marked with a dash. Summary information is presented for the full cohort, unless the cohort size is listed.

**Table 4:** Detailed demographic details of the test set.

TEST SET (n = 471)	DLBS	SALD	MMALS	PREVENT-AD
n	85	123	73	190
Age	54.06 (20.90)	43.71 (17.17)	47.93 (14.25)	63.10 (4.60)
Men	55.97 (21.14)	45.30 (17.90)	44.00 (16.26)	63.61 (5.03)
Women	53.07 (20.90)	42.85 (16.82)	49.86 (12.90)	62.89 (4.40)
Sex	56F; 29 M	80F; 43 M	49F; 24 M	134F; 56 M
TIV (cm^3^)	1038.43 (116.52)	1056.82 (99.65)	1038.76 (111.95)	1007.48 (92.82)
Education (years)	16.46 (2.62)	–	15.36 (2.02)	15.69 (3.36)
% of +FH (for the available cohorts)	–	–	41.10%	100%
*APOE* Status (count)	n = 70	–	n = 71	n = 190
e2/e2	3		0	0
e2/e3	7		3	14
e3/e3	44		41	107
e4/e2	4		3	6
e4/e3	12		22	58
e4/e4	0		2	5
BMI (kg/m^2^)	–		24.90 (3.82)	22.20 (3.86)
MOCA	–		29.03 (1.09)	28.03 (1.54); n = 189
MMSE	28.59 (1.25)		29.07 (1.00)	–
Systolic BP	–	–	118 (15.67); n = 43	129.94 (16.52)
Diastolic BP	–	–	74.58 (8.77); n = 43	75.28 (8.99)
Physical Activity	–	–	–	n = 180
Low Exercise				103
High Exercise				77

TIV = Total Intracranial Volume; +FH = positive family history of AD; BMI = Body Mass Index; MOCA = Montreal Cognitive Assessment; MMSE = Mini Mental State Examination; BP = Blood Pressure. Note: The PREVENT-AD was solely used in the Test Set. Education level is obtained as the total number of years of education in the DLBS and PREVENT-AD cohorts, and total number of years in proportion to the degree obtained for the MMALS cohort. Values in brackets are in standard deviation. Information that was not collected/reported by the datasets are marked with a dash. Summary information is presented for the full cohort, unless the cohort size is listed.

The model was then trained on the training set using a tuning grid search at different values of alpha with 10-fold cross-validation, repeated 10 times, to determine the optimal lambda (‘shrinkage’) for each alpha value, to predict age from brain cortical thickness measures, while minimizing the root mean squared error (RMSE) between true and predicted age (see Supplementary Appendix for detailed methods).

The brain structural features from the training set were then used to build an elastic net regularized regression model using the glmnet (Friedman et al., 2010) package (R 3.6.3, R Core Team, 2013). Covariates in the analysis were intracranial volume (ICV; a sum of cerebrospinal, white matter, and grey matter volume), Sex, Site, and Euler number (a measure of MRI image quality control [QC] provided by FreeSurfer; Rosen et al., 2018). The continuous variables (i.e., brain structural measures, ICV, Euler number) were z-score standardized, while Sex and Site were treated as categorical factor variables. In total, 82 predictors were used in the age prediction model: 64 cortical thickness features, 14 subcortical measures, and 4 covariates (Supplementary Table 6 for full list of predictors). We ran the training model on the full cohort which were individuals with scans that were successfully preprocessed and with Euler numbers that fell within 3 standard deviations of the mean. We also performed rigorous QC of this larger cohort and ran the training model on individuals who passed strict QC (Supplementary Appendix and Supplementary Tables 3-5).

#### 2.3.2 Model testing

The trained elastic net model was then applied to the testing dataset to predict participants’ ages from their brain structural features (in addition to the covariates of interest) using the parameters estimated from the training dataset. Continuous variables were z-score standardized, whereas Sex and Site were treated as categorical factor variables. The testing set included the remaining 30% of the uFH cohort (n = 251) and the +FH cohort (n = 220) from the PREVENT-AD and the MMALS cohorts.

#### 2.3.3 The impact of Sex differences in family history and APOE status on BAG

BAG (i.e., Estimated Age – Chronological Age) was used as a measure of brain structural health. The BAG represents the individual deviation of chronological age from the typical aging trajectory (Franke and Gaser, 2019) and is used to denote neurobiological aging processes. Negative values represent a younger appearing brain and positive values represent an older appearing brain compared to the norm (Cole et al., 2019, Elliott et al., 2019, but see Vidal-Piñeiro et al., 2021). For example, if a participant has a predicted age of 70 years but their actual age is 80 years, their BAG number is −10, which means that their brain is considered to be 10 years younger than the normative brain of an 80-year-old. If their actual age is 60 years, then their BAG is +10 reflecting an older appearing brain relative to the norm. Thus, positive BAG numbers would indicate an older appearing brain, whilst all negative BAG numbers represent a younger appearing brain.

First, we used a multiple regression model to examine whether Sex interacted with FH status in the effects of BAG on middle-aged and older adults (≥ 40 years) in the test set (n = 376; 259F, 117 M). This cohort consisted of participants in the MMALS and PREVENT-AD cohort for which FH status was known. We further excluded younger adults from this analysis because of the possibility that these effects might be driven artificially by younger adults, none of which had +FH status. We then ran a multiple regression model to investigate whether *APOE* status contributed to BAG in individuals with a FH of AD. This secondary analysis consisted of a subsample of participants with the e3/e3 (*-APOE4*) or e3/e4 (*+APOE4*) *APOE* alleles (n = 191, 136F, 55 M) because there were insufficient data to test for the effect of the other alleles.

#### 2.3.4 Association of modifiable factors with BAG

Multiple regression analyses were conducted to determine whether modifiable factors —Blood Pressure, BMI (n = 173, 124F, 49 M), and Physical Activity (n = 141, 98F, 43 M) —interacted with AD risk factors—FH status, *APOE* status—to influence structural brain aging on the PREVENT-AD cohort. One outlier was removed for the analysis with BMI and two outliers were removed for the analysis with Physical Activity.

In all our post-hoc regression analyses (see Supplementary Appendix for regression models), we included the participant’s chronological age as a covariate in the models to account for the observed bias in brain age models, which tends to overestimate BAG in younger adults and underestimate the gap in older adults.(de Lange and Cole, 2020, Le et al., 2018, Liang et al., 2019, Niu et al., 2020, Smith et al., 2019). Analyses were performed in R version 3.6.3 (R Core Team, 2013) and *P-*values<0.05 were considered statistically significant.

## 3 Results

### 3.1 Brain age model performance

**Fig. 1:**
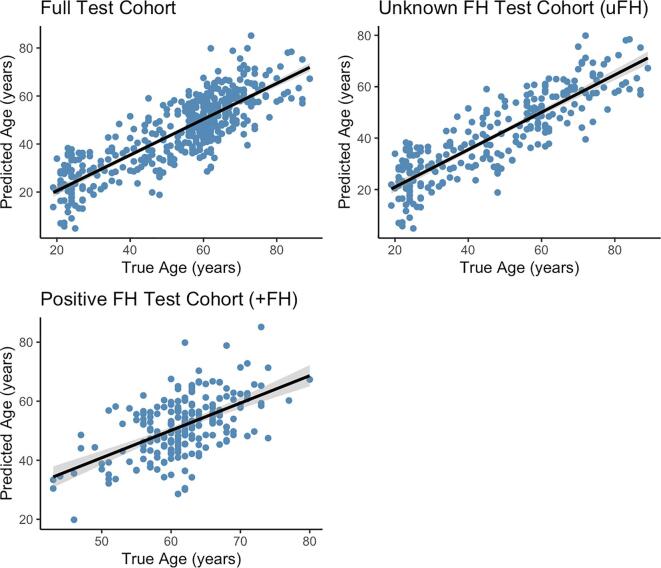
The correlation between true age and predicted age of the full cohort in the test set (r = 0.83, p < 0.05) and split by FH status: participants with unknown FH (uFH; r = 0.86, p < 0.05) and with positive FH (+FH; r = 0.56, p < 0.05) of AD. The shaded areas represent the 95% confidence interval.

The trained model yielded an optimal shrinkage parameter (lambda) of 0.24, which was obtained with a mixing parameter (alpha) of 0.55 yielding a RMSE of 10.24 years. The mean absolute error of prediction was 8.22 years. Cross-validation results gave a correlation between predicted age and chronological age of r = 0.86 *(P* < 0.05) in the uFH cohort, and r = 0.56 (*P* < 0.05) in the +FH cohort. The model explained 74% of the age variance (R^2^) in the uFH cohort and 31% in the +FH cohort (Fig. 1). The trained model was more reliably able to predict age from brain structure in the uFH cohort than the +FH cohort (see Fig. 1 and Supplementary Table 7 for the top 25 ROI features of age prediction). The results for the more rigorous QC cohort were similar to the full cohort model results (Supplementary Appendix), so we report the full cohort model here.

### 3.2 Association of FH status and Sex with BAG

An ANCOVA used to test the explicit interaction between FH status and Sex revealed a significant interaction, i.e., F(3, 371) = 2.97, *P* = 0.03. Post-hoc tests (using the Bonferroni correction to adjust for multiple comparisons) indicated that women with +FH had a significantly smaller negative BAG than men with +FH (*P* = 0.008). No sex differences occurred in BAG in the uFH cohort (*P* = 1.00). This analysis did not include the younger adult cohort used in the test set because we did not have any +FH young participants. Since this is the only post-hoc analysis that is conducted on datasets with varying Scanner Sites, we re-ran this analysis adjusting for Scanner Site and found that the Sex*FH interaction remained significant, F(3, 369) = 2.94, *P* = 0.03. To ensure that the effects were not driven by the middle-aged participants, we also re-ran the analysis on the older adult cohort alone (60 years and older, n = 225), finding that the interaction between Sex and FH Status remained significant (*P* = 0.03). Since participants in the uFH cohort comprise of negative or unknown FH status, all subsequent post-hoc analyses focus on participants with a known +FH status.

### 3.3 Association of APOE status and Sex on BAG

To understand whether the Sex effect in +FH on BAG was related to *APOE* effects, we used an ANCOVA to evaluate whether *APOE* status contributed to the greater BAG in women than men in a subset of participants. One participant had missing *APOE* information from the +FH cohort, so they were removed from further analyses. Participants were removed from the post-hoc analyses due to limited numbers in the *APOE* groups which are 14 e2/e3 carriers, 8 e2/e4 carriers, and 6 e4/e4 carriers. Thus, of the 219 +FH participants with *APOE* status information, only the 191 participants with *APOE* status of e3/e4 or e3/e3 were used in post-hoc analyses.

The explicit interaction between *APOE* status (e3/e3 and e3/e4 groups) and Sex on the effect of BAG was tested in the +FH subsample that had either the *-APOE4* or *+APOE4* genotype (n = 191), revealing a Sex*APOE status effect on BAG, F(3, 186) = 3.19, *P* = 0.02. Bonferonni-corrected post-hoc tests revealed that *+APOE4* women had a smaller negative BAG than *+APOE4* men (*P* = 0.046), and a significant sex difference was not observed in the *-APOE4* group (*P* = 0.08). Given the possibility that gene-environment interactions can influence BAG, next we tested whether modifiable AD risk factors contributed to this Sex*APOE status relationship.

### 3.4 Association of modifiable factors and APOE status in the effects of BAG

**Fig. 2:**
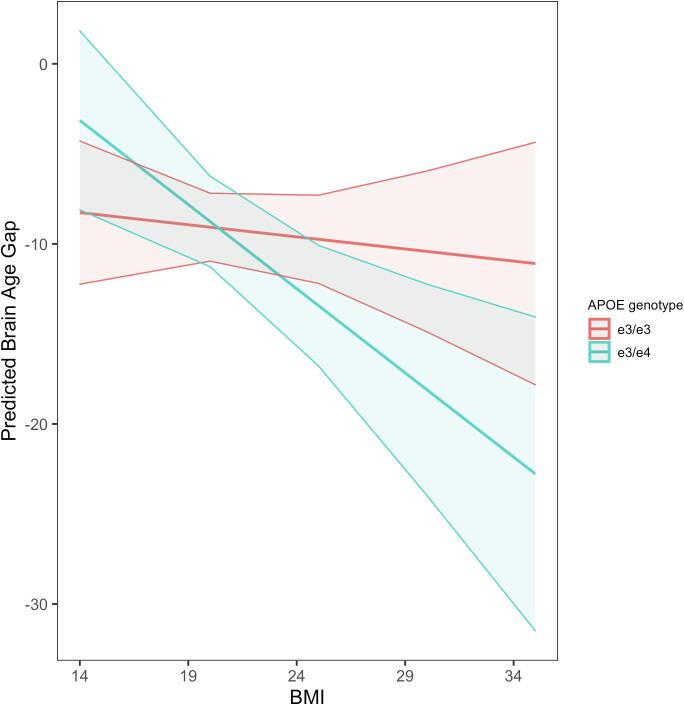
The significant interaction between *APOE* status and BMI on the predicted BAG, which was driven by women specifically. This interaction revealed that women with an *+APOE4* genotype and a higher BMI showed a larger negative BAG than women with an *–APOE4* genotype and a higher BMI. The shaded areas represent the 95% confidence interval.

We conducted multiple regression analyses to test the interaction of three modifiable factors (Blood Pressure, BMI, Physical Activity) with *APOE* status and Sex effects on BAG. The models were not significant when Blood Pressure (systolic, diastolic) was tested, so this modifiable factor was not used in further post-hoc analyses. We observed a significant BMI*APOE status interaction, (β = −3.21 [SE, 1.52]; *P* = 0.04), which revealed that individuals with a higher BMI and *+APOE4* risk showed a larger negative BAG than individuals with a higher BMI but no *APOE4* risk. This interaction remained significant when the model was tested in women only (β = −2.85 [SE, 1.43]; *P* = 0.049; Fig. 2) but the model was not significant when tested in men only, suggesting that the observed interaction was driven by women.

**Fig. 3:**
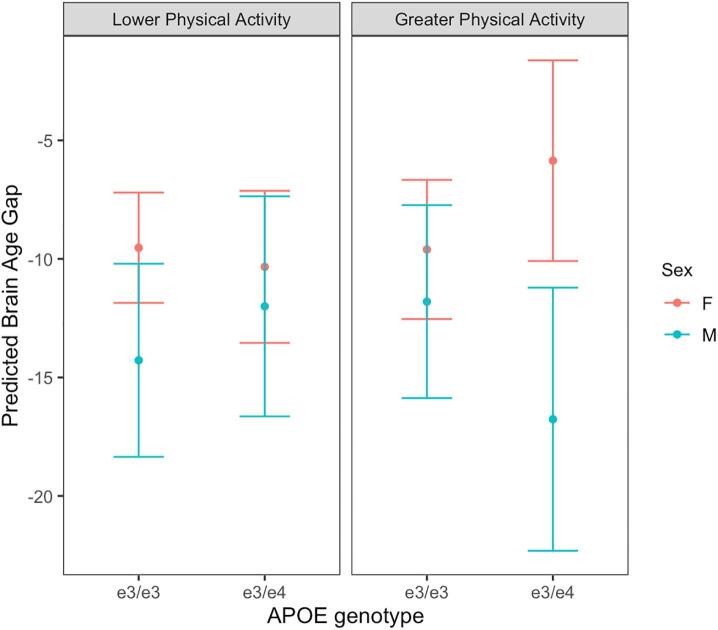
The significant interaction between *APOE* status, Sex, and Physical Activity on the predicted BAG. Men with the *+APOE4* genotype who engaged in greater physical activity had a larger negative brain age gap than women with the *+APOE4* genotype who engaged in greater physical activity. Error bars represent the 95% confidence intervals.

A significant three-way interaction between *APOE* status, Sex, and Physical Activity was also observed (β = −11.79 [SE, 5.71]; *P* = 0.04, Fig. 3). A Tukey’s post-hoc test revealed that in *+APOE4* adults, greater physical activity was related to a larger negative BAG in men than in women (*P* = 0.049). No other significant differences were found. A logistic regression revealed no significant effect of BMI on the level of Physical Activity (*P* > 0.05), demonstrating that these two variables were not correlated with each other.

Logistic regressions were also completed to determine that there was no effect of *APOE* status on Age, Sex, ICV, Physical Activity and Education within the three test datasets (DLBS, MMALS, PREVENT-AD). In addition, we collapsed across datasets to determine if these variables played a role on the full test cohort. There were no effects of *APOE* status on Age, Sex, and ICV effects on the full test cohort (n = 286). There was also no effect of BMI on the PREVENT-AD and MMALS collapsed cohort (n = 227; DLBS cohort was excluded because this dataset did not collect the BMI of their participants).

## 4 Discussion

A growing body of research indicates that BAG (i.e., Estimated Age – Chronological Age ) is a sensitive metric for detecting early signs of AD (Gaser et al., 2013, Löwe et al., 2016). In the current study, we used elastic net regression to predict brain age in uFH and +FH adults to test the hypothesis that there were sex differences in the effect of unmodifiable AD risk factors on BAG. The more negative a BAG score, the younger the brain age. Participants in the +FH cohort had a mean negative BAG and thus, a healthy brain age, but differences emerged in the degree of negative BAG within the +FH cohort. Consistent with our hypothesis, we found that middle-aged and older women with unmodifiable risk factors (+FH and *+APOE4*) had an older brain age than men with equivalent risk factors, indicating that women with unmodifiable risk factors have brain anatomy that deviates more than men’s from the normative aging trajectory. In addition, we found that modifiable risk factors, BMI and physical activity, contributed to how far +FH and *+APOE4* women’s BAG deviated from the uFH normal aging trajectory. In the following sections, we discuss these findings in greater detail.

Our findings demonstrate that women with non-modifiable risk factors of AD (+FH and *+APOE4*) had an ‘older’ appearing brain than men. This is consistent with studies demonstrating that both +FH and *+APOE4* was associated with advanced structural brain aging in cognitively normal adults (Bendlin et al., 2010, Donix et al., 2010, Espeseth et al., 2008). While *+APOE4* confers greater risk for women than men (Altmann et al., 2014, Breitner et al., 1999, Bretsky et al., 1999, Farrer et al., 1997, Mortensen and Hogh, 2001, Payami et al., 1996), no studies to date have shown sex differences in +FH risk on the effects of BAG, a potential indicator of accelerated aging. It is important to note that, on average, the +FH cohort had negative BAG values. However, the extent of how negative their BAG values were differed according to the risk factors assessed. Thus, our analysis supports the cognitively healthy status of participants (as shown by neuropsychological scores), while also demonstrating that some groups had healthier brains than others.

Independent of *APOE* status, +FH status has previously been associated with increased vascular and inflammatory markers (van Exel et al., 2009), which at chronic levels, has been linked to greater brain atrophy and increased risk of age-related pathologies (Lunetta et al., 2007, Maggio et al., 2006). However, these studies did not segregate their analyses by sex, which might mask underlying sex-related biological mechanisms associated with +FH status. For example, one study found that postmenopausal women with AD, for whom +FH status was common, had significantly higher serum levels of estrone and its precursor, androstenedione, compared to controls (Cunningham et al., 2001); but, no significant group differences in levels of testosterone or estradiol were observed. Women with estrogen replacement therapy were excluded from this study, and the AD group had significantly less body mass than the control group. Since these effects remained after adjusting for a variety of demographic and biological factors (e.g., age, BMI, cortisol, alcohol intake), the authors suggest that the difference could be attributed to abnormal levels of sex steroid production in AD, which may interact in complex ways with +FH status. Clearly, a closer examination of the specific estrogen subtype and differences in the menopause transition will clarify the role of menopause and estrogen levels on regulating adiposity and inflammatory processes in women with +FH.

In addition, sociocultural factors may accompany +FH. For example, in +FH families, women are often caregivers for individuals with dementia, placing them at greater risk for developing dementia themselves (Norton et al., 2010). Caregivers of dementia patients suffer a greater degree of stress than other caregivers, with physiological and immune consequences such as high levels of stress hormones, reduced immune function, and greater cardiovascular health challenges (e.g., coronary heart disease; Carter et al., 2012). Thus, a complex interplay of sex-specific hormones, sociocultural factors, and AD risk factors may contribute to different brain-aging trajectories among +FH individuals. Future work should consider sex differences in genetic and non-genetic factors associated with +FH status on brain health and risk for AD conversion.

Our study also brings some clarity to the debate about the role of late-life BMI in brain aging and its downstream contributions for dementia risk (Bolzenius et al., 2015, Shaw et al., 2018), by highlighting the importance of considering *APOE* genotype by sex. BMI interacted with *APOE* status in +FH women; in women with *+APOE4* genotype, those with greater BMI benefited from a lower BAG than those with lower BMI. Women with AD risk factors who convert to dementia have been shown to be more vulnerable to weight loss, particularly early in the AD transition prior to clinical symptoms (Johnson et al., 2006, White et al., 1998). Since *+APOE4* women are more likely to convert to AD than *+APOE4* men, higher BMI might protect against weight loss associated with transitioning to dementia. Higher late-life BMI has been linked to a reduced risk of converting from MCI to AD, a protective effect that did not persist in the presence of rapid weight loss (Bell et al., 2017). This protective effect of higher baseline BMI did persist in the presence of *+APOE4*, suggesting an interaction of BMI and *APOE* status (Bell et al., 2017). At midlife when women experience menopause-related loss of 17β-estradiol from the ovaries, fat tissue becomes a primary source of estrogens (Simpson, 2003)*.* Thus, higher late-life BMI might be associated with greater circulating estrogen which might be neuroprotective, particularly in post-menopausal women in which the ovaries cease to become the primary source of estrogen release. Importantly, it is critical to investigate whether different sources of estrogen exposure (e.g., age at menarche and menopause, time since menopause, and duration of hormone replacement therapy) and the role of female-specific hormonal transitions, such as parity, differentially contributes to brain aging in women (de Lange et al., 2020a, de Lange et al., 2020b).

In addition, sex differences in physical activity revealed that *+APOE4* women did not benefit from greater physical activity as much as *+APOE4* men did. This is contrary to the consensus that physical activity is beneficial in protecting brain structural health in all older adults, but that there may be a critical interaction with *APOE* genotype. Greater physical activity was less protective to brain age in *+APOE4* women than it was to *+APOE4* men who engaged in greater physical activity, which might be consistent with the idea that preserving body weight may be more protective to women at greater AD risk. Importantly, gendered patterns in physical activity may contribute to how exercise benefits women and men (see Subramaniapillai et al., 2021 for discussion of gendered contribution of lifestyle factors). For example, more than men, women report they exercise for weight loss and toning (Crawford and Eklund, 2016, Furnham et al., 2002, Hsiao and Thayer, 1998, McDonald and Thompson, 1992, Prichard and Tiggemann, 2005, Tiggemann and Williamson, 2000), while more than women, men report exercising for social, competitive, and pleasure purposes (Markland and Hardy, 1993, Silberstein et al., 1988). Thus, women engaging in exercise for weight loss in particular may experience advanced brain aging particularly regarding interactions with the *+APOE4* genotype.

## 5 Limitations

The limited availability of demographic information in some of the datasets prevented us from conducting comprehensive post-hoc analyses or really knowing how many participants with a possible family history of AD existed in the uFH data set. Unfortunately, the uFH cohort consists of both participants for whom FH is known to be negative and those for whom no FH status is collected. Regardless of this limitation, we still found a significant interaction between Sex and FH Status, demonstrating greater BAG (less negative) in the +FH cohort compared to the uFH cohort.

Although only 31% of the entire +FH cohort had *+APOE4,* the effects were still robust compared to the uFH cohort. Unfortunately, the small number of participants in the other *APOE* carrier status groups was not sufficient to perform meaningful inferential statistics, so only participants with e3/e4 or e3/e3 genotypes were analysed in the present study. Our cohort consisted of a larger proportion of women than men. To address this potential caveat, we split the data into train and test sets according to Age Group and Sex so that the numbers are represented across train and test sets within the uFH cohort. Moreover, when we ran the models separately in women and men, we found similar results (see Supplementary eFigure 2). Perhaps most importantly, these numbers of women and men in each cohort reflect the actual sex differences in AD with women carrying twice the burden at every age starting at 45. Future work should explore these effects in a much larger cohort of participants with the full breadth of *APOE* carrier distributions. Finally, it is critical that future longitudinal studies of neurocognitively healthy middle-aged and older +FH adults be conducted to determine whether this cohort continue to follow a healthy brain-aging trajectory, or transition to a more pathological one.

The current findings suggest a complex relationship between non-modifiable and modifiable risk factors, and patterns of brain aging in women and men. Although non-modifiable risk factors (i.e., +FH and *+APOE4*) contribute to an older brain age in women compared to men, modifiable factors have the potential to mitigate this relationship by decreasing the BAG. Future work should endeavour to measure modifiable factors in clinical trials to investigate this relationship in order to make appropriate conclusions on the effects of modifiable and risk factors on BAG in men and women.

## Authorship Contribution Statement

**Sivaniya Subramaniapillai:** Conceptualization, Methodology, Formal analysis, Writing - original draft, Writing - review & editing, Visualization, Funding acquisition. **Sricharana Rajagopal:** Methodology, Validation. **Jamie Snytte:** Validation, Writing - review & editing. **A. Ross Otto:** Methodology, Formal analysis, Writing - review & editing. **PREVENT-AD Research Group:** Investigation, Data Creation & Curation. **Gillian Einstein:** Writing - review & editing. **M. Natasha Rajah:** Conceptualization, Methodology, Writing - review & editing, Supervision, Funding acquisition.
