# Supplementary material for: Sex differences in brain aging among adults with family history of Alzheimer’s disease and APOE4 genetic risk
Source: Neuroimage Clin. 2021 Mar 10;30:102620. doi: 10.1016/j.nicl.2021.102620 (PMC8065341; doi:10.1016/j.nicl.2021.102620)
Supplement: Supplementary data 1 [file mmc1.docx]

**SUPPLEMENTARY**

**Supplementary Appendix. Supplementary Methods**

Neuroimaging dataset details

These four datasets are specifically used because they contain cognitively healthy aging cohorts. Although criteria across datasets varied slightly in terms of how cognitive health was obtained, the datasets used robust neuropsychological tests (e.g., Mini-Mental State Examination, Montreal Cognitive Assessment, and/or Clinical Dementia Rating Scale) to ensure participants did not have dementia at time of testing. In addition, all participants in these cohorts were highly educated.

***Dallas Lifespan Brain Study (DLBS):***

Wave 1 of the DLBS collection consists of 315 healthy adults (aged 20-89 years) collected from the Dallas-Fort Worth community (2008-2014). Participants were recruited through traditional methods of advertisement, including media advertisement and flyers. Participants were native English speakers and right-handed. Participants also underwent health history screening via a health questionnaire as well as telephone and personal interviews. All participants included in the study were screened for cardiovascular issues, drug/alcohol abuse, and neurological and psychiatric disorders. Participants were excluded if they sustained a head injury with loss of consciousness for more than 10 minutes or had any MRI safety contraindications. All participants scored 26 or greater on the MMSE. Participants received a structural MRI with DTI, three task-based functional MRI scans, and a resting-state scan. T1 volumes were obtained using a Phillips Achieva 3T with an MPRAGE sequence (scan parameters: TR = 8.1 ms; TE = 3.7 ms; flip angle = 12°; voxel size=1×1×1 mm). See Supplementary Table 2 for summary of MRI parameter details across datasets. See Kennedy et al., 2015 and Chan, Park, Savalia, Petersen, & Wig, 2014 for more information.

***Southwest University Adult Lifespan Dataset (SALD)***

The SALD consists of 494 healthy adults (aged 19-80 years) who received extensive testing (structural MRI, resting-state fMRI, and basic phenotype information) at the Brain imaging Center of Southwest University (Chongqing, China; 2010-2015). Participants were recruited through traditional methods of advertisement, including online advertisements and distribution of leaflets*.* The cohort was recruited at or near the university: young adults were enrolled at Southwest University; middle-aged adults were largely staff at the university, and the remainder of the participants were recruited from communities near the campus. Participants were excluded if they met the MRI-related exclusion criteria (e.g., claustrophobia, metallic implants, Meniere’s Syndrome, history of fainting in the last 6 months), or their profiles included psychiatric or neurological disorders, psychiatric drug use within 3 months prior to scanning, or head trauma. Participants were also excluded if they were pregnant. Since the Wei et al. (2018) paper does not report how participants’ cognitive status was obtained, the authors were contacted to determine the source of this information: all participants had to have an MMSE score of 25 and greater (i.e., cognitively healthy). T1 structural images were acquired using a 3T Siemens Trio with an MPRAGE sequence (TR = 1900 ms, TE = 2.52 ms, flip angle = 90°, 256 x 256 resolution matrix, voxel size = 1 x 1 x 1 mm^3^). See Supplementary Table 2 for summary of MRI parameter details across datasets. See Wei et al., 2018 for more information on the cohort characteristics.

***Montreal Memory and Aging Lifespan Study (MMALS)***

The MMALS consists of 172 healthy adults (aged 19-76 years) who received neuropsychological testing and administration of a medical screening questionnaire to determine eligibility to participate in the MRI study. Screening questionnaires included the Mini-Mental Status Exam (MMSE, inclusion cut-off score > 26, Folstein, Folstein, & McHugh, 1975) the Beck Depression Inventory (BDI, inclusion cut-off < 15, Beck & Steer, 1987), and the American National Adult Reading Test (NART, inclusion cut-off ≤ 2.5 SD, Strauss, Sherman, & Spreen, 2006). Exclusion criteria from the medical screening questionnaire also included self-reported history of neurological insult, psychiatric illness, substance abuse, smoking > 50 cigarettes/day, diabetes, BMI > 30, or current diagnosis of high cholesterol and/or high blood pressure that had been treated for less than six months or left untreated.

If participants met the aforementioned study inclusion/exclusion criteria, they were invited to Session 2 of the study to participate in the fMRI scanning session. If participants met the inclusion criteria for the MRI study, they were scheduled for the second session, which included acquisition of a structural T1w MRI, and functional MRI scans obtained while subjects performed an episodic memory task. A high-resolution T1w structural image was acquired from each subject using a 3D gradient echo MPRAGE sequence (TR = 2300 msec, TE = 2.98 msec, flip angle 9°, 176 1mm sagittal slices, 1x1x1 mm voxels, FOV 256 mm^2^). See Supplementary Table 2 for summary of MRI parameter details across datasets.

***Pre-symptomatic Evaluation of Experimental or Novel Treatments for Alzheimer’s Disease (PREVENT-AD)***

The PREVENT-AD (Douglas Mental Health Institute, Montreal) is a longitudinal cohort consisting of 385 cognitively unimpaired older individuals with a family history of Alzheimer’s disease (i.e., AD-like dementia in at least one parent or multiple siblings (>=2)). Information about participants’ family history of AD was obtained using either the Cache County Study questionnaire or a compelling report from an experienced clinician (Tremblay-Mercier et al., 2020). Participants were recruited between September 2011 and November 2017 through distribution of flyers throughout Montreal and surrounding cities, in addition to media (TV, radio, newspapers) advertisement. Participants were eligible for the study if they were 60 years or older. Individuals aged 55-59 years were recruited if they were <15 years from the age of their relatives at symptom onset. Sufficient fluency in English and/or French was necessary to participate in the study. Participants had no history of major neurological or psychiatric disease, and were examined annually using clinical and cognitive tests, blood tests and MRI. 191 participants between the ages of 55 to 80 and CDR = 0, with baseline structural MRI were included in the present study.

Several assessments were completed to ensure participants’ cognitive health. First, cognitive health was assessed using the Montreal Cognitive Assessment (MoCA) and the Clinical Dementia Rating (CDR). If participants scored lower than normal on these measures (MoCA ≤ 26 or CDR>0), a neuropsychologist then conducted an evaluation to determine if scores bordering the cut-off criteria were circumstantial. Second, participants also completed a ~30-minute MRI session to screen for structural brain disease. Finally, all study participants had to have a study partner available to ensure their cognitive status (i.e., provide information on their daily functioning). Thus, study participation required intact cognition and stable general health. Participants were additionally excluded if they were on medication (e.g., acetyl-cholinesterase inhibitors, anticoagulants, NSAIDs, opiates, memantine or other prescription-approved cognitive enhancements). Individuals were also excluded on the basis of current alcohol, barbiturate or benzodiazepine abuse/dependence. Participants were also excluded if they had significant hypertension (unless controlled medically), anemia, or significant liver or kidney disease. See Tremblay-Mercier et al., 2020 for more information.

T1w structural image was acquired from each subject using 3D gradient echo MPRAGE sequence (TR = 2300 ms, TE = 30 ms, flip angle 9^o^, 176 1mm sagittal slices, 1x1x1 mm voxels, FOV 256mm^2^). See Supplementary Table 2 for summary of MRI parameter details across datasets.

***Genotyping***

*APOE* status was determined through genotyping of venous blood samples, collected for three datasets: MMALS, DLBS, and PREVENT-AD.

Supplementary Table 1.

| Dataset | Genotyping Procedure |
| --- | --- |
| DLBS | - Venous blooded collected into EDTA-anticoagulated tubes - Genomic DNA isolated: 50-70 xx of DNA isolated from 2 mL of whole blood - *APOE* genotypes determined by real-time PCR using TaqMan probes (Applied Biosystems, Inc., Foster City, CA) - See Rodrigue et al., 2012 for more information |
| MMALS | - Genomic DNA extracted from whole blood using FlexiGene DNA kit (Qiagen, Ontario, Canada) - *APOE* genotypes determined using Sequenom iPLEX Gold Assay technology (Genome Quebec Innovation Centre, Quebec, Canada; Agena-Bioscience, 2015) - See Rajah et al., 2017 for more details. |
| PREVENT-AD | - Genomic DNA extracted from 200 *μl* whole blood using a QIASymphony apparatus and the DNA Blood Mini QIA Kit (Qiagen, Valencia, CA, USA). - *APOE* genotype determined using the PyroMark Q96 pyrosequencer (Qiagen) - See Tremblay-Mercier et al., 2020 for more information |

***Quality Control (QC) measures***

T1w scans were visually inspected for image artefacts by two independent raters (S.S. and J.S.). Using a quality control protocol (Bedford et al., 2020), we assigned scans a rating of 1 if they were free from artefacts and a rating of 4 if there was significant ringing and blurring. Scans were removed if they had a mean score greater than 2.5. Disagreement between images were discussed between raters until consensus was reached (if ratings were higher than 1 point difference) or an average of the ratings were produced (if off by 0.5).

***Preprocessing of the structural MRIs***

*Cortical thickness (CT) measurements*

Following QC of images, T1w scans were preprocessed using the minc-bpipe-library pipeline (<https://github.com/CobraLab/minc-bpipe-library>), which first registers the T1w images to the MNI space, followed by an iterative N4 algorithm to correct for acquisition-related inhomogeneities in image intensity (Tustison et al., 2010). The output of the pipeline generates a bpipe mask for each participant. Images were then submitted to the CIVET automated preprocessing pipeline (CIVET 2.1.10), which is a tissue classification algorithm that segments the MRI structural image into white matter (WM), grey matter (GM), cerebral spinal fluid (CSF), and background (Zijdenbos, Forghani, & Evans, 2002). The output of running the MRI images through CIVET is a measurement of cortical thickness (the distance between inner and outer gray matter surfaces) at 81, 924 points across the cortex. The parameters used in CIVET were a smoothing kernel of 30 mm using the laplace method for thickness estimates, a 12-parameter linear registration, and partial volume estimates to define deep sulci (June et al., 2005). CIVET also enables parcellation of the original 81, 924 points across the cortex using the Desikan-Killiany-Tourville (DKT) atlas, which averaged the cortical measurements for each subject for a set of 64 regions.

QC of images was performed at several steps along the pre-processing stages. First, the bpipe pipeline output generated brain masks which were then visually inspected (by S.S. and C.R.) to determine if the brain mask fully encompassed the cerebrum, while excluding the cerebellum. Second, the CIVET pipeline produced “verify” images generated from the pipeline which enabled raters (S.S. and C.R.) to visually inspect the grey and white matter classification for any errors (e.g., mixing of GM as WM). Images were ranked by the two raters as pass, warn, or fail (see CIVET protocol; Bedford et al., 2020). Lastly, the surface-to-surface interactions of the CIVET output was visually inspected to assess any tissue classification errors (mixing of GM with WM); images with surface-to-surface interactions greater than 100 per hemisphere were discarded from the final analysis. QC performed at these four steps resulted in exclusion of data from 618 participants in the QC cohort. The primary analysis included the full cohort, and only excluded participants from the analysis if the MRIs failed to be processed by the preprocessing pipelines (bpipe, and CIVET) which resulted in the removal of 63 participants. See Supplementary Tables 3 and 4 Supplementary for the different steps of the QC preprocessing pipeline for the full and QC cohorts, respectively.

*Subcortical Structures*

MRI images were also processed using FreeSurfer 6.0 (Fischl et al., 2002) to segment and obtain volumes of subcortical areas. In total, segmentation of cortical thickness and subcortical measures yielded a total of 78 structural brain imaging features (64 cortical thickness measures and 14 subcortical measures). In the full cohort, images were removed if FreeSurfer failed to generate a Euler number for the participant (N = 5) or if the Euler number fell outside of ± 3SD. This resulted in 22 participants that were removed (N = 1067) from the full cohort and 9 participants that were removed from the QC cohort (N = 526). Supplementary Table 4 provides a summary of the final QC cohort demographics.

*Elastic Net Model*

Elastic net is a generalized linear model specifically designed to handle a larger number of features relative to the size of the cohort. This model uses a mixture of LASSO and ridge regression by adjusting the elastic net mixing parameter α, with range of α ∈ [0,1]. The L1 regularization in LASSO (α = 1) penalty forces many features to have zero coefficient values while the ridge (α = 0) regression penalty (i.e., L2 regularization) does not eliminate features entirely, but instead, minimizes the value of the coefficients of correlated predictors towards each other, producing similar values between correlated variables. Values in between are a mixture of ridge and LASSO, where α < 0.5 performs more like ridge on the model, while α > 0.5 performs more like LASSO on the model. Regularization enables a grouping effect where strongly correlated predictors tend to share similar coefficient weightings. This method is a modification of the OLS regression which penalizes features that have multicollinearity. This is primarily done through a regularization (penalty/lambda) parameter which shrinks coefficients that are correlated with each other to have similar values. The penalty λ parameter controls the strength of the regularization (Friedman, Hastie, & Tibshirani, 2010).

*Analyses on Quality Control (QC) Cohort*

We trained a separate model on the passed quality control images (N = 526, train set = 290, test set = 236; see Supplementary Table 5) to determine whether including versus excluding failed quality control imaging data contributed to significant differences in model performance. Using the QC cohort, we first removed images that fell outside of ± 3SD of the Euler number. We preserved the training vs. test split of the full model cohort to assess deviation metrics of brain age on the training set when using the full cohort compared to the same participants used in the QC cohort. The analysis revealed optimal tuning parameters of alpha = 1.00 and lambda = 0.24. The root mean square error was 9.47 years and the mean absolute error of prediction was 7.58 years. Cross-validation results gave a correlation between predicted age and chronological age of r = 0.86 (p < 0.05) in the –FH cohort, and r = 0.56 (p<0.05) in the +FH cohort. These results are almost identical to the correlations found in the larger cohort.

**Summary of Modifiable Factors**

*Cardiovascular Health Assessment*

Blood pressure was measured using an automatic sphygmomanometer (Connex ProBP 3400; Welch Allyn) as participants were seated. BMI was assessed using the participants weight over height (kg/m^2^).

*Physical Activity*

Participants’ level of physical activity level was originally categorized under one of 4 options: “1 = light intensity”, “2 = regular light intensity”, “3= regular moderate intensity”, “4 = regular high intensity”. To enhance cohort distributions across physical activity groups, we combined participants into two physical activity groups: “Lower physical activity” (options 1 + 2) and “Higher physical activity” (options 3 + 4).

*Cultural Effects on Brain Age Gap Estimation*

Given that the SALD dataset was the only cohort that consisted of participants from China, whereas the other three datasets were collected from Canada and the USA, it is possible that cultural differences may be driving the brain age effects. We performed a multiple linear regression to determine if there were any significant differences in BAG based on the dataset, where Age was used as a covariate: BAG ~ Dataset + Age. There was no significant effect of the Dataset, suggesting no one neuroimaging dataset was driving the BAG effects.

**Post-hoc Analyses (conducted in R 3.6.3)**

**Analysis 1:**

anova(lm(Brain Age ~ FH:Sex + Age.z))

- Tested explicit differences in FH*Sex (with no lower order main effects)

**Analysis 2:**

anova(lm(Brain_Age ~ *APOE*:Sex + Age.z))

- Only considered +FH participants with e3/e3 or e3/e4 so final sample

**Analysis 3:**

Blood Pressure:

1. lm(Brain Age ~ Systolic BP**APOE**Sex + Age)
   - Model ns
2. lm(Brain Age ~ Diastolic BP**APOE**Sex + Age)
   - Model ns

BMI

1. lm(Brain Age ~ BMI**APOE**Sex + Age)
2. Within Sex analysis:
   1. In females: lm(Brain Age ~ BMI**APOE* + Age)
   2. In males: lm(Brain Age ~ BMI**APOE* + Age)

Physical Activity:

1. lm(Brain Age ~ Physical Activity**APOE**Sex + Age)

Logistic Regression: Is BMI related to PA?

glm(PA ~ BMI + Age + Sex)

Supplementary Table 2: MRI summary parameters across neuroimaging datasets

|  | Scanner | Sequence | Scan Parameters |
| --- | --- | --- | --- |
| MMALS | 3T Siemens | MPRAGE | TR = 2300 ms  TE = 2.98 ms  flip angle = 9°  voxel size = 1x1x1 mm^3^  256 x 256 matrix size  Scan duration (sec): 312 |
| PREVENT-AD | 3T Siemens | MPRAGE | TR = 2300 ms  TE = 30 ms  flip angle = 9°  voxel size = 1x1x1 mm^3^  256 x 256 matrix size  Scan duration (sec): 312 |
| DLBS | 3T Philips Achieva | MPRAGE | TR = 8.1 ms  TE = 3.7 ms  flip angle = 12°  voxel size = 1×1×1 mm^3^  Scan Resolution: 256 x 256  Scan duration (sec): 237 |
| SALD | 3T Siemens Trio | MPRAGE | TR = 1900 ms  TE = 2.52 ms  flip angle = 90°  voxel size: 1 x 1 x 1 mm^3^  Scan Resolution: 256 x 256  Scan duration (sec): NA |

Supplementary Table 3. Full Cohort

| Database | Total N (raw) | bpipe run | CIVET run | Final Cohort |
| --- | --- | --- | --- | --- |
| MMALS | 172 | 0 | 3 | 169 |
| PREVENT-AD | 195 | 0 | 4 | 191 |
| DLBS | 315 | 1 | 28 | 287^#^ |
| SALD | 471 | 1 | 27 | 443 |
| TOTAL | 1153 | 2 | 62 | 1090 |

Note: Numbers in columns between ‘Total N’ and ‘Final n’ represent the total number of failed images.

#1 participant removed for not having relevant demographic information

Supplementary Table 4. QC Cohort

| Database | Total N | T1 QC | B bpipe run | bpipe QC | CIVET run | QC surface-surface  intersections | QC verify (<1) | Final n |
| --- | --- | --- | --- | --- | --- | --- | --- | --- |
| C MMALS | 172 | 39 | 0 | 4 | 2 | 1 | 1 | 125 |
| PREVENT-AD | 195 | 106 | 0 | 0 | 1 | 0 | 1 | 87 |
| DLBS | 315 | 169 | 1 | 1 | 10 | 14 | 4 | 116^#^ |
| SALD | 471 | 236 | 0 | 2 | 7 | 13 | 7 | 207 |
| TOTAL | 1153 | 550 | 1 | 7 | 19 | 28 | 13 | 535 |

Note: Numbers in columns between ‘Total N’ and ‘Final n’ represent the total number of failed images.

#1 participant removed for not having relevant demographic information

Supplementary Table 5. QC demographic details

| QC Cohort | Train | Test 1: Healthy cohort | Test 2: FH cohort (PREVENT-AD cohort & MMALS) |
| --- | --- | --- | --- |
| n | 290 | 123 | 113 (n = 26 MMALS) |
| Age (SD) | 44.30(17.51) | 44.21 (18.55) | 59.96 (6.38) |
| Sex | 103 (36%) M  187 (64%) F | 43 (35%) M  80 (65%) F | 27 (24%) M  86 (76%) F |
| Age Group | 124 (43%) YA  100 (34%) MA  66 (23%) OA | 54 (43%) YA  40 (33%) MA  29 (24%) OA | 48 (42%) MA  65 (58%) OA |
| Total Intracranial Volume, cm^3^ (SD) | 1056.79 (107.88) | 1052.13 (103.77) | 1012.78 (93.98) |
| Age Group: Male | 28 OA M  27 MA M  47 YA M | 9 OA M  13 MA M  21 YA M | 18 OA M  9 MA M |
| Age Group: Female | 38 OA F  73 MA F  77 YA M | 20 OA F  27 MA F  33 YA F | 47 OA F  39 MA F |

Supplementary Table 6: List of features used in age prediction model

| **Feature Variable** |
| --- |
| CaudalAnteriorCingulate.L_CT |
| CaudalAnteriorCingulate.R_CT |
| CaudalMiddleFrontal.L_CT |
| CaudalMiddleFrontal.R_CT |
| Cuneus.L_CT |
| Cuneus.R_CT |
| EntorhinalCortex.L_CT |
| EntorhinalCortex.R_CT |
| FusiformGyrus.L_CT |
| FusiformGyrus.R_CT |
| InferiorOccipitalCortex.L_CT |
| InferiorOccipitalCortex.R_CT |
| InferiorParietal.L_CT |
| InferiorParietal.R_CT |
| InferiorTemporal.L_CT |
| InferiorTemporal.R_CT |
| Insula.L_CT |
| Insula.R_CT |
| IsthmusCingulateGyrus.L_CT |
| IsthmusCingulateGyrus.R_CT |
| LateralFrontalOpercularis.L_CT |
| LateralFrontalOpercularis.R_CT |
| LateralFrontalOrbitalis.L_CT |
| LateralFrontalOrbitalis.R_CT |
| LateralFrontalTriangularis.L_CT |
| LateralFrontalTriangularis.R_CT |
| LateralOrbitofrontal.L_CT |
| LateralOrbitofrontal.R_CT |
| LingualGyrus.L_CT |
| LingualGyrus.R_CT |
| MedialOrbitofrontal.L_CT |
| MedialOrbitofrontal.R_CT |
| MiddleFILLING.L_CT |
| MiddleFILLING.R_CT |
| MiddleTemporal.L_CT |
| MiddleTemporal.R_CT |
| ParacentralGyrus.L_CT |
| ParacentralGyrus.R_CT |
| Parahippocampal.L_CT |
| Parahippocampal.R_CT |
| Pericalcarine.L_CT |
| Pericalcarine.R_CT |
| PostcentralGyrus.L_CT |
| PostcentralGyrus.R_CT |
| PosteriorCingulate.L_CT |
| PosteriorCingulate.R_CT |
| PrecentralGyrus.L_CT |
| PrecentralGyrus.R_CT |
| Precuneus.L_CT |
| Precuneus.R_CT |
| RostralAnteriorCingulate.L_CT |
| RostralAnteriorCingulate.R_CT |
| RostralMiddleFrontal.L_CT |
| RostralMiddleFrontal.R_CT |
| SuperiorFrontalGyrus.L_CT |
| SuperiorFrontalGyrus.R_CT |
| SuperiorParietal.L_CT |
| SuperiorParietal.R_CT |
| SuperiorTemporal.L_CT |
| SuperiorTemporal.R_CT |
| SupramarginalGyrus.L_CT |
| SupramarginalGyrus.R_CT |
| TransverseTemporal.L_CT |
| TransverseTemporal.R_CT |
| Left-Hippocampus |
| Left-Caudate |
| Left-Putamen |
| Left-Pallidum |
| Right-Caudate |
| Right-Hippocampus |
| Right-Thalamus-Proper |
| Right-Putamen |
| Left-Thalamus-Proper |
| Right-Accumbens-area |
| Right-Pallidum |
| Right-Amygdala |
| Left-Amygdala |
| Left-Accumbens-area |
| average_euler |
| ICV |
| Sex |
| Site |

Note: In black are values derived from CIVET 2.1.0. In orange, are values from FreeSurfer. In purple are the covariates used.

Supplementary Table 7: Top 25 brain structural features for brain age estimation model sorted by absolute weight

| Predictor | Raw | Absolute Value |
| --- | --- | --- |
| Right-Thalamus-Proper | -3.45 | 3.45 |
| Right-Putamen | -2.83 | 2.83 |
| Left-Thalamus-Proper | -2.82 | 2.82 |
| TransverseTemporal.R_CT | -2.76 | 2.76 |
| MiddleTemporal.L_CT | -2.67 | 2.67 |
| Right-Accumbens-area | -2.54 | 2.54 |
| LingualGyrus.R_CT | -2.25 | 2.25 |
| ParacentralGyrus.L_CT | -2.21 | 2.21 |
| MedialOrbitofrontal.R_CT | -2.21 | 2.21 |
| InferiorOccipitalCortex.R_CT | 2.11 | 2.11 |
| LateralOrbitofrontal.R_CT | 2.02 | 2.02 |
| TransverseTemporal.L_CT | -1.85 | 1.85 |
| CaudalMiddleFrontal.L_CT | -1.74 | 1.74 |
| Right-Pallidum | 1.67 | 1.67 |
| Pericalcarine.R_CT | -1.65 | 1.65 |
| SuperiorParietal.R_CT | 1.39 | 1.39 |
| MiddleFILLING.L_CT | -1.21 | 1.21 |
| MiddleFILLING.R_CT | -1.15 | 1.15 |
| EntorhinalCortex.R_CT | 1.14 | 1.14 |
| LateralFrontalTriangularis.R_CT | -1.12 | 1.12 |
| InferiorTemporal.L_CT | 1.12 | 1.12 |
| ParacentralGyrus.R_CT | -1.10 | 1.10 |
| IsthmusCingulateGyrus.L_CT | 1.08 | 1.08 |
| SuperiorParietal.L_CT | 1.04 | 1.04 |
| SuperiorFrontalGyrus.R_CT | -1.01 | 1.01 |

Supplementary Figure 1.

Note: Top 20 coefficients from the three training models (from left to right: 1) full sample model, 2) female only sample model, and 3) male only sample model) that were plotted (colours representing the coefficient values, with greater numbers representing regions which the model attributed greater weight). The two additional training samples were run to determine if models performed differently in women only vs and men only. Our results demonstrated considerable overlap of regions of interest between men and women that was shared with the full sample model and similar model parameters that were generated after training the different samples, justifying the use of using the full sample model in the primary analysis. Female sample training model (n = 380): alpha = 0.55 and lambda = 0.24, RMSE = 10.06, R^2^= 0.71, MAE = 8.02, correlation between predicted age and chronological age of test set (n =319): 0.79, p<0.05. Male Sample Model (n = 216): alpha = 1, lambda= 0.25, RMSE = 10.81, R^2^ = 0.68, MAE = 8.62, correlation between predicted age and chronological age of test set (n = 152): 0.82, p < 0.05.

**Supplementary References**

Beck, A. T., & Steer, R. A. (1987). Manual for the revised Beck depression inventory. *San Antonio, TX: Psychological Corporation.*

Bedford, S. A., Park, M. T. M., Devenyi, G. A., Tullo, S., Germann, J., Patel, R., … Chakravarty, M. M. (2020). Large-scale analyses of the relationship between sex, age and intelligence quotient heterogeneity and cortical morphometry in autism spectrum disorder. *Molecular Psychiatry*, *25*(3), 614–628. https://doi.org/10.1038/s41380-019-0420-6

Chan, M. Y., Park, D. C., Savalia, N. K., Petersen, S. E., & Wig, G. S. (2014). Decreased segregation of brain systems across the healthy adult lifespan. *Proceedings of the National Academy of Sciences of the United States of America*, *111*(46), E4997–E5006. https://doi.org/10.1073/pnas.1415122111

Fischl, B., Salat, D. H., Busa, E., Albert, M., Dieterich, M., Haselgrove, C., … Dale, A. M. (2002). Whole brain segmentation: Automated labeling of neuroanatomical structures in the human brain. *Neuron*, *33*(3), 341–355. https://doi.org/10.1016/S0896-6273(02)00569-X

Folstein, M. F., Folstein, S. E., & McHugh, P. R. (1975). “Mini-mental state”. A practical method for grading the cognitive state of patients for the clinician. *Journal of Psychiatric Research*, *12*(3), 189–198. https://doi.org/10.1016/0022-3956(75)90026-6

Friedman, J., Hastie, T., & Tibshirani, R. (2010). Regularization paths for generalized linear models via coordinate descent. *Journal of Statistical Software*, *33*(1), 1–22. https://doi.org/10.18637/jss.v033.i01

Jennifer Tremblay-Mercier, Cécile Madjar, Samir Das, Stephanie O.M. Dyke, Pierre Étienne, Marie-Elyse Lafaille-Magnan, Pierre Bellec, D. Louis Collins, M. Natasha Rajah, Veronique D. Bohbot, Jeannie-Marie Leoutsakos, Yasser Iturria-Medina, Justin Kat, R, J. C. S. B. & the P.-A. R. G. (2020). Creation of an Open Science Dataset from PREVENT-AD, a Longitudinal Cohort Study of Pre-symptomatic Alzheimer’s Disease.

June, S. K., Singh, V., Jun, K. L., Lerch, J., Ad-Dab’bagh, Y., MacDonald, D., … Evans, A. C. (2005). Automated 3-D extraction and evaluation of the inner and outer cortical surfaces using a Laplacian map and partial volume effect classification. *NeuroImage*, *27*(1), 210–221. https://doi.org/10.1016/j.neuroimage.2005.03.036

Kennedy, K. M., Rodrigue, K. M., Bischof, G. N., Hebrank, A. C., Reuter-Lorenz, P. A., & Park, D. C. (2015). Age trajectories of functional activation under conditions of low and high processing demands: An adult lifespan fMRI study of the aging brain. *NeuroImage*, *104*, 21–34. https://doi.org/10.1016/j.neuroimage.2014.09.056

Strauss, E., Sherman, E., & Spreen, O. (2006). A Compendium of Neuropsychological Tests: Administration, Norms, and Commentary. Oxford University Press.

Tustison, N. J., Avants, B. B., Cook, P. A., Zheng, Y., Egan, A., Yushkevich, P. A., & Gee, J. C. (2010). N4Itk. *IEEE Trans Med Imaging*, *29*(6), 1310–1320. https://doi.org/10.1109/TMI.2010.2046908.N4ITK

Wei, D., Zhuang, K., Ai, L., Chen, Q., Yang, W., Liu, W., … Qiu, J. (2018). Data Descriptor: Structural and functional brain scans from the cross-sectional Southwest University adult lifespan dataset. *Scientific Data*, *5*, 1–10. https://doi.org/10.1038/sdata.2018.134

Zijdenbos, A. P., Forghani, R., & Evans, A. C. (2002). Automatic “pipeline” analysis of 3-D MRI data for clinical trials: Application to multiple sclerosis. *IEEE Transactions on Medical Imaging*, *21*(10), 1280–1291. https://doi.org/10.1109/TMI.2002.806283
